# Whole-Genome Sequencing of Pathogenic Bacteria—New Insights into Antibiotic Resistance Spreading

**DOI:** 10.3390/microorganisms9122624

**Published:** 2021-12-19

**Authors:** Andrey Shelenkov

**Affiliations:** Central Research Institute of Epidemiology, Rospotrebnadzor, 111123 Moscow, Russia; shelenkov@cmd.su

In recent years, the acquisition of antimicrobial resistance (AMR) by both pathogenic and opportunistic bacteria has become a major problem worldwide, which was already noticed as a global healthcare threat by the World Health Organization [1]. The spread of multidrug-resistant bacteria, especially those producing extended-spectrum β-lactamases (ESBLs), represents a major challenge in clinical settings [2]. According to some estimations, the number of deaths caused by antibiotic resistant bacteria will exceed those caused by all cancer types by 2050 if the existing trend continues [3].

However, the mechanisms of antibiotic resistance acquisition and spreading within and across bacterial species cannot be revealed by traditional phenotypic or PCR-based genotypic tests, although such analyses provide valuable information and are still extensively used in molecular epidemiology studies [4]. Whole-genome sequencing (WGS) is currently attracting an increasing amount of attention since it allows accurate, rapid and cost-effective acquisition of the data regarding the presence of specific antibiotic resistance genes, as well as the determination of various isolate and plasmid replicon classification and typing markers. In addition to traditional molecular tools such as multilocus sequence typing (MLST) or serotyping, which are available for most clinically important bacterial species [5], WGS provides an easy access to additional sequence-based typing techniques which have significantly higher specificity and resolution. These include capsular polysaccharide (KL) and lipooligosaccharide outer core (OCL)-based schemes for *Acinetobacter baumannii* [6,7] and *Klebsiella pneumoniae* [8], which can still be implemented using traditional molecular methods, but become dramatically easier to perform using WGS, as well as even more complex and precise typing schemes such as the ones based on a hybrid KL/OCL/MLST approach [9], CRISPR sequences [10], frequency-domain sequence characteristics [11], or core genome MLST (cgMLST) [12], which become cost-ineffective or even impossible to perform without WGS data.

Thus, WGS provides additional tools for bacterial isolate typing with better specificity and higher resolution, which can be easily implemented in epidemiological surveillance protocols to provide additional valuable data regarding the spread and transfer of the given species or genes on the level of a particular hospital, region, country or even worldwide.

Recent advances in this field have already given rise to the development of ‘genomic epidemiology’ approaches which combine various bioinformatics methods for tracing the spread of bacterial and viral pathogens. Such approaches have been successfully used for outbreak investigations [13,14,15] and for studying the variety and predominance of pathogenic bacteria isolates belonging to different species on the scale of a particular hospital [16,17,18], region [19] or country [20,21,22].

However, typing the isolates and monitoring their spread and/or predominance in a particular setting will not per se provide the insights into the ways of resistance spreading and acquisition, although the isolates belonging to some MLST-based sequence types were associated with higher AMR in various bacterial species [23,24,25]. Since AMR is often conferred by either resistance gene acquisition through external mobile elements or mutations in chromosomal genes [26], then the information concerning accurate chromosome and, especially, plasmid structures could provide substantial benefits for AMR investigations. For this purpose, WGS becomes indispensable, in particular, when different sequencing methods (short- and long-read) are applied simultaneously to the same isolate. Plasmid reconstruction using long-read sequencing data can provide essential information regarding the mechanisms of antibiotic resistance acquisition and the way it spreads across different species and world regions.

In addition, portable long-read sequencers such as MinION (Oxford Nanopore Technologies, Oxford, UK) could provide rapid identification of resistance determinants with a potential application in clinical analyses and antimicrobial therapy corrections [27,28,29].

Thus, WGS provides an efficient way of studying the presence and transfer of antibiotic resistance determinants with a potential application in clinical settings.

In view of the aforesaid, the aim of this special issue was to additionally highlight the role of WGS in studying the ways and mechanisms of AMR acquisition, which was already noticed by respected organizations, such as the EUCAST [30] and NIHR [31]. Kuleshov et al. [32] applied the power of WGS to investigate the plasmid-mediated colistin resistance of *Salmonella enterica* isolates from Russia. Using long-read sequencing, they determined the precise locations of *mcr-1* and *mcr-9* resistance genes in *S. enterica* plasmids. Khezri et al. [33] performed a comprehensive analysis of different mono- and hybrid (short- and long-read) assembly tools, which allowed them to conclude that the utilization of two sequencing technologies provides better sequence resolution and allows the refined determination of AMR and virulence genes’ presence and location in *Escherichia coli* and *K. pneumoniae* clinical isolates. The other planned papers are supposed to provide additional examples of WGS application to AMR investigations in both clinical and foodborne pathogens.

We believe that the information provided in this special issue will facilitate the investigations of AMR spreading mechanisms and will contribute to the development of novel approaches to epidemiological surveillance and infection prevention for various bacterial pathogens.
